# *Bartonella* Infections in Cats and Cat Fleas in Lithuania

**DOI:** 10.3390/pathogens10091209

**Published:** 2021-09-17

**Authors:** Miglė Razgūnaitė, Indrė Lipatova, Algimantas Paulauskas, Birutė Karvelienė, Vita Riškevičienė, Jana Radzijevskaja

**Affiliations:** 1 Faculty of Natural Sciences, Vytautas Magnus University, K. Donelaičio Str. 58, LT-44248 Kaunas, Lithuania; migle.razgunaite@vdu.lt (M.R.); indre.lipatova@vdu.lt (I.L.); jana.radzijevskaja@vdu.lt (J.R.); 2 Faculty of Veterinary Medicine, Lithuanian University of Health Sciences, Tilžės Str. 18, LT-47181 Kaunas, Lithuania; birute.karveliene@lsmuni.lt (B.K.); vita.riskeviciene@lsmuni.lt (V.R.)

**Keywords:** pet cats, stray cats, cat fleas, *Bartonella* *henselae*, *Bartonella clarridgeiae*, 16S–23S rRNA ITS

## Abstract

*Bartonella* are vector-borne parasitic bacteria that cause zoonotic infections in humans. One of the most common infections is cat-scratch disease caused by *Bartonella henselae* and *Bartonella clarridgeiae*. Cats are the major reservoir for these two species of bacteria, while cat fleas are vectors for the transmission of infection agents among cats. The aim of the present study was to investigate the presence of *Bartonella* infections in stray and pet cats and in cat fleas in Lithuania. Blood samples were taken from 163 cats presented in pet clinics and animal shelters. A total of 102 fleas representing two species, *Ctenocephalides felis* and *Ctenocephalides canis*, were collected from 12 owned cats that live both outdoors and indoors. *Bartonella* DNA in samples was detected using a nested PCR targeting the 16S–23S rRNA intergenic spacer (ITS) region. *Bartonella* DNA was detected in 4.9% (8/163) of the cats and 29.4% (30/102) of the fleas. Sequence analysis of the ITS region showed that the cats and fleas were infected with *B. henselae*, *B. clarridgeiae* and *Bartonella* sp., closely related to *B. schoenbuchensis*. This study is the first report on the prevalence and molecular characterization of *Bartonella* spp. in cats and cat fleas in Lithuania.

## 1. Introduction

Domestic pets are susceptible to infection by various species of *Bartonella* and can play a role in human infection. Cats (*Felis catus*) are considered the main reservoir of three zoonotic *Bartonella* species: *B. henselae*, *B. clarridgeiae* (both of which can cause cat-scratch disease) and *B. koehlerae* (a causative agent of endocarditis in humans). Cat-scratch disease (CSD) is the best-known infection caused by *Bartonella* bacteria [1,2,3,4]. This zoonosis has a worldwide distribution, but is more commonly detected in warmer climate zones. In northern temperate zones, it occurs more frequently between August and October, usually in humid, warm locales [5,6,7]. Cats can also be the accidental host of *B. quintana*, *B. vinsonii* subsp. *berkhoffii*, *B. elizabethae*, *B. bovis* (*ex weissii*), *B. volans*-like, *B. grahamii*, *B. rochalimae* and *B. washoensis* [1,3,8,9,10]. The cat flea, *Ctenocephalides felis*, is the main vector of *B.* *henselae* and a potential vector of *B. clarridgeiae* and *B. koehlerae* [11,12,13]. *Bartonella quintana*, *B. rochalimae*, *B. elizabethae*, *B. grahamii*, and *B. alsatica* have been also detected in cat fleas [13,14,15,16,17,18]. *Bartonella vinsonii* subsp. *berkhoffii* has been amplified from *Pullex* spp. fleas [3].

Cats are in close contact with humans, many of them even sleeping in the same bed as their owners [19]. It is also common for pet cats to have access to outdoor areas where there is a high likelihood of exposure to ectoparasites or pathogens. Given the high frequency of very close contact between cats and their owners, as well as between cats and other domestic animals, cats can play an important role in the maintenance and transmission of zoonotic agents to humans. The transmission of the causative agent of CSD from felines to humans has been reported all over the world [20,21,22,23].

There is limited information about CSD and other *Bartonella* infections in humans in Lithuania. The oldest known cases of a lice-borne disease caused by *B. quintana* were reported in soldiers in Napoleon’s Grande Armée in Vilnius [24]. Specific antibodies to *B. henselae* in human serum samples were detected by serologic testing in 2006 in Lithuania’s National Public Health Surveillance Laboratory [25]. To date, two cases of CSD in Lithuania have been described in the literature, in 2008 and 2014, respectively. The first case of CSD-associated encephalopathy in a 16-year-old boy was confirmed based on clinical manifestations and positive serology in 2007 [26]. The other known clinical case of cat-scratch neuroretinitis (ocular bartonellosis) was reported in 2014 in a patient in a Lithuanian hospital [27].

One of the most important methods of zoonosis prevention is an investigation of the prevalence of pathogens in their vectors and reservoir hosts [3,28,29]. However, there is still a lack of studies on vector-borne zoonotic pathogens from cats and their ectoparasites. In view of the emergence of zoonotic *Bartonella* infections, plus the ubiquity and abundance of cats and their close association with humans and the absence of information about occurrences of *Bartonella* spp. in the Lithuanian population of domestic cats and their ectoparasites, the aim of this study was to investigate the presence of *Bartonella* in pet and stray cats and cat fleas and to characterize *Bartonella* strains by PCR and sequence analysis of the 16S–23S rRNA intergenic species region (ITS).

## 2. Results

Blood samples were collected from 163 cats, 90 (55.2%) of which were male and 73 female (44.8%). The age of the cats ranged from three months to nineteen years (with a median of four years). Cats were divided into two age groups: young kittens <1 year old (n = 20) and adults >1 year old (n = 143). Based on their health status, cats were divided into apparently healthy (57.7%; n = 94) and sick (42.3%; n = 69).

A total of 102 fleas representing two species, *Ct. felis* (n = 92) and *Ctenocephalides canis* (n = 10), were collected from twelve owned domestic cats. Flea infestation ranged from one to thirty-four fleas per cat. The fleas were 27.5% (n = 28) male and 72.5% (n = 74) female.

*Bartonella* DNA was detected in 4.9% (8/163) of cat samples and 29.4% (30/102) of flea samples (Table 1). Among the PCR-positive cats, three (37.5%) were young kittens, and five (62.5%) were adults. *Bartonella* spp. DNA was detected in 27.8% (5/18) of the stray cats and 2.1% (3/145) of the pet cats (χ^2^ = 22.68, *p* < 0.05). Clinical signs of *Bartonella* infection were observed in 50% (n = 4) of the infected cats: 12.5% of the animals had lethargy, 12.5% diarrhea, 12.5% visual impairment, and 12.5% altered general blood test parameters (Table 2).

*Bartonella*-positive fleas were obtained from six owned cats (one young kitten and five adults). The percentage of fleas infected with *Bartonella* spp. varied among cat hosts from 6.3 to 50% (Table 3). Both flea species harbored *Bartonella* pathogens. Of the thirty *Bartonella*-positive fleas, 25 (83.3%) were *Ct. felis* and five (16.7%) were *Ct. canis* (χ^2^ = 2.26, *p* > 0.05). *Bartonella* DNA was detected in 17.9% (5/28) of male fleas and 33.8% (25/74) of female fleas (χ^2^ = 2.48, *p* > 0.05) (Table 1).

**Figure 1:**
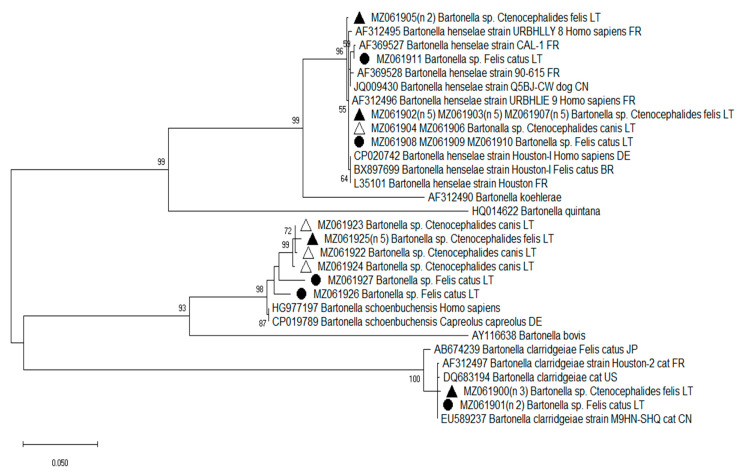
Phylogenetic tree for the partial ITS region of *Bartonella* spp. inferred using the maximum-likelihood method and Tamura 3-parameter model with 1000 bootstrapping replications. Samples sequenced in the present study are marked with ● (*Felis catus*), ▲ (*Ctenocephalides felis*) and ∆ (*Ctenocephalides canis*). The number of samples represented by the sequence is given in parentheses (n x).

A total of 38 *Bartonella* 16S–23S rRNA ITS region sequences (eight from cats and 30 from fleas) were analyzed. Sequence analysis demonstrated that the cats and fleas were infected with *B. henselae* (23 sequences; MZ061902–MZ061911), *B. clarridgeiae* (five sequences; MZ061900–MZ061901) and *Bartonella* sp., closely related to *B. schoenbuchensis* (ten sequences; MZ061922–MZ061927) (Table 1; Figure 1). The phylogenetic tree showed three well-supported clusters (Figure 1). One cluster contained the human- and cat-associated species *B. henselae*, *B. koehlerae* and *B. quintana*. Another cluster consisted of ruminant-associated *Bartonella* spp. (*B. schoenbuchensis* and *B. bovis*). The third group included different *B. clarridgeiae* strains. The ITS sequences of *B. henselae*, *B. clarridgeiae* and *Bartonella* sp. derived from cats and fleas in this study were heterogenic. Among the Lithuanian *B. henselae* isolates, three ITS genotypes (two in cats and two in fleas) with five variable nucleotides were detected (Table 4). Two cat specimens and three *Ct. felis* flea specimens harbored two different *B. clarridgeiae* genotypes, respectively (differing at two nucleotide positions). The *B. henselae* and *B. clarridgeiae* ITS region sequences obtained in this study were 98–100% identical to the corresponding sequences available in GenBank. Twenty *B. henselae* ITS sequences obtained in the cats and fleas were 100% identical to the URBHLIE 9 strain (which had previously been isolated from patients with endocarditis) and differed from the Houston-1 strain (identified in both animals and humans and involved in CSD) by one nucleotide substitution (C→G) at position 66 in the analyzed sequences [30]. Two other *B. henselae* ITS sequences obtained in this study were specific to Lithuanian samples and were closely related to CAL-1 and URBHLLY 8 strains (differing by one and four nucleotides, respectively) previously isolated from human patients in France (Table 4; Figure 1).

Genetic heterogeneity was found to be higher among *Bartonella* sequences more closely related to *B. schoenbuchensis*, with DNA similarity values from 95 to 96% and six genotypes identified. *Bartonella* isolates derived from cats and from *Ct. canis* and *Ct. felis* fleas were species-specific and differing by thirty-two nucleotide positions. *Bartonella* sequences (MZ061926 and MZ061927) obtained in two cats were 96–98% identical (differing at eight and sixteen nucleotides, respectively) to the corresponding sequence of the *B. schoenbuchensis* strain detected in humans (HG977197). *Ctenocephalides canis* and *Ct. felis* fleas harbored *Bartonella* genotypes (MZ061922–MZ061925), which shared 94–98% similarity in the ITS region sequence (differing from ten to fourteen nucleotide positions) with *B. schoenbuchensis* isolates derived from human and roe deer (HG977197 and CP019789) (Figure 1).

## 3. Discussion

This study is the first report of the presence of *Bartonella* infections in cats and cat fleas in Lithuania. Two causative agents of cat-scratch disease, *B. henselae* and *B. clarridgeiae*, were identified in the cats and fleas, and *B. henselae* was found to be more common than *B. clarridgeiae*.

In Europe, serological prevalence rates of *Bartonella* infections in cats are high in European Mediterranean countries, where the temperature and humidity are favorable for flea infestation, and range from 0% in Norway to 71.4% in Spain. PCR-confirmed cases of feline bartonellosis have rarely been reported, with the molecular prevalence of *Bartonella* infection in cats ranging from 0% in north-east Germany to 83.5% in Italy [3].

*Bartonella* species appear to be highly adapted to one or a few mammalian reservoir hosts. Infection in these hosts is characterized by long-lasting intraerythrocytic bacteraemia [6,31]. The clinical spectrum of the infection in felines has not been fully investigated, but naturally infected animals seem to be healthy carriers. In cases when symptoms appear, they are most commonly mild clinical signs. Experimentally infected cats have shown signs of lymphadenopathy, myalgia and transient fever with lethargy and anorexia during febrile periods [1]. Young and stray cats are more likely to be carriers of the pathogen than older (>1 year) or pet cats [1,32,33]. The results of the present study showed that 50% of the infected cats had certain medical conditions, which can be associated with *Bartonella* infection (lethargy, diarrhea, visual impairment, and altered general blood test parameters).

The prevalence of *Bartonella* infection was significantly higher in the stray cats than in the pet cats (27.8 and 2.1%, respectively; χ^2^ = 22.68, *p* < 0.05), and cats in the adult age group were more frequently infected than young kittens (62.5 and 37.5%, respectively; χ^2^ = 4.98, *p* < 0.05). Among the 90 male and 73 female cats tested, three (3.3%) and five (6.9%) specimens, respectively were infected. However, the detected difference in the prevalence of infection between males and females was not statistically significant (χ^2^ = 1.07, *p* > 0.05). Although previous studies have demonstrated that male and younger cats are more likely to be infected with *Bartonella* pathogens due to their potentially more aggressive nature [34,35], such data were not obtained in the present study.

The pathogens can be transmitted among cats or from cats to humans by scratches or bites by infected animals or cat fleas, and through the feces of infected fleas [31,36]. In Europe, due to their widespread distribution, fleas are especially important in the transmission of *Bartonella* species from pets to humans. *Ctenocephalides felis* is the most common flea species found on cats, followed by *Ct. canis* [18,37]. In the present study, two flea species—cat flea *Ct. felis* and dog flea *Ct. canis*—were collected from owned (outdoor/indoor) domestic cats living in suburban and rural areas. *Ctenocephalides felis* was more abundant (90.92% of the total; collected from twelve cats) than *Ct. canis* (9.8% of the total; only found on three cats). *Bartonella* spp. were detected in 27.2% (25/92) of the *Ct. felis* and 50% (5/10) of the *Ct. canis* fleas. Sequence analysis of positive samples showed that both species of fleas harbored *B. henselae*, while *B. clarridgeiae* was only detected in *Ct. felis*. These results confirmed that *Ct. felis* and *Ct. canis* fleas may play an important role in the transmission of zoonotic *Bartonella* species in Lithuania. *Bartonella henselae* and *B. clarridgeiae* in cat fleas have also been reported in other studies conducted worldwide [2,12,13,14,15,16,17,18,38].

Unexpectedly in this study, *Bartonella* genotypes closely related genetically to *B. schoenbuchensis* were detected in cats and in both flea species. These *Bartonella* genotypes have not yet been documented as infecting cats in Europe. *Bartonella schoenbuchensis* strains are associated with ruminants and usually transmitted by deer keds (*Lipoptena* spp.) [39,40,41,42]. In Lithuania, strains closely related to *B. schoenbuchensis* have been detected in deer keds *Lipoptena cervi* and *Lipoptena fortisetosa* (GenBank: MT873590–MT873595) [43]. Wild and domestic ruminants have also been found to harbor *Bartonella capreoli*, *Bartonella chomelii*, *Bartonella melophagi* and *Bartonella bovis* [40,44,45]. *Bartonella schoenbuchensis*, *B. capreoli* and *B. chomelii* (which have been isolated from roe deer, red deer and cattle) are genetically and biologically close species and are members of the same *Bartonella* clade [46]. *Bartonella bovis* (formerly *Bartonella weissii*) is commonly detected in cattle [44,47,48,49] and can cause endocarditis in infected animals [50]. However, this species was originally isolated from four domestic cats from Utah and Illinois (USA) and, together with *B. henselae*, *B. clarridgeiae*, and *B. koehlerae,* was reported as the fourth *Bartonella* species found to infect cats in North America [1,51]. Breitschwerdt et al. suggested that the detection of *B. weissii* in both cats and cattle may reflect an unusual evolutionary adaptation for this particular *Bartonella* species [47].

Due to their high interspecies variability and low intraspecies variability, several protein-encoding genes, such as those encoding cell division protein (ftsZ), citrate-synthase (gltA), haem-binding protein (pap31), heat-shock protein (groEL), NADH dehydrogenase gamma subunit (nuoG), riboflavin synthase (ribC), RNA polymerase beta-subunit (rpoB), 17-kDa antigen, 35-kDa protein and the 16S rRNA gene, have been used to infer evolutionary relationships between *Bartonella* strains and identify genotypes [2,52,53,54,55,56]. 16S–23S rRNA ITS sequences have also been confirmed as a useful tool for phylogenetic analyses at the interspecies level and for *Bartonella* species subtyping [30]. Molecular characterization of *B. henselae* strains demonstrates the genotypic heterogeneity of *B. henselae* in patients with CSD. Based on 16S rRNA sequence analysis, two main genotypes of *B. henselae* in human patients with cat-scratch disease have been described and correspond to two serotypes: Houston-1 and Marseille [17,56,57].

The present study demonstrated genetic heterogeneity among *B. henselae* and *B. clarridgeiae* strains circulating in cats and fleas in Lithuania. The *B. clarridgeiae* sequences obtained showed 99–100% identity to *B. clarridgeiae* detected in cats in France, China, Japan and the USA (AF312497, EU589237, AB674239 and DQ683194). The *B. henselae* ITS sequences showed high identity (99–100%) with *B. henselae* strains isolated from patients infected with CSD in France (AF312496) and with the *B. henselae* Houston-I genotype detected in cats and humans in Germany, France and Brazil (CP020742, BX897699 and L35101). The *B. henselae* Marseille genotype is known to be dominant in cat populations in western Europe, the western United States and Australia, while the Houston genotype is dominant in Asia [58,59]. However, the prevalence of different *B. henselae* genotypes may vary among cat populations in the same country. In France, the Marseille genotype has been shown to be dominant in central France, while the Houston genotype is dominant in the south of France [32]. There is evidence that these two genotypes vary in their zoonotic potential: Houston has been associated with more severe clinical manifestations than those induced by Marseille [60].

It has been suggested that *Bartonella* infections may persist in both cats and fleas. Although most cats infected with *B. henselae* and *B. clarridgeiae* are usually asymptomatic, they serve as reservoirs of these bacteria and transmit the infection to humans. As reservoir hosts for *B. henselae*, cats can be subclinically infected for months and even years [3]. The present findings suggest a potential risk of humans acquiring CSD causative agents in Lithuania and underline the need for the routine diagnosis of *Bartonella* infections in Lithuania’s cat population.

## 4. Materials and Methods

### 4.1. Sample Collection

Blood samples were taken from 163 domestic cats in two veterinary clinics (n = 145) and two animal shelters (n = 18) in Kaunas (Central Lithuania) in 2016–2018. Feline blood samples were taken from the cephalic vein into tubes containing EDTA and kept at +4 or −20 °C until DNA isolation. The clinical symptoms, sex and age of the cats were recorded during a physical examination. Information about outdoor access or the flea infestation status of cats collected in veterinary clinics and animal shelters was not available.

Fleas were collected from 12 owned domestic cats that live both outdoors and indoors in two Lithuanian districts (Kaunas and Joniškis) in 2015–2016. The fleas collected from each cat were placed in separate 1.5 mL tubes with 70% ethanol, and kept at +4 °C until investigation. Flea species were identified by morphological criteria [37].

### 4.2. DNA Extraction and PCR Amplification

DNA from the blood of the cats was extracted using a GeneJet Whole Blood Genomic DNA Purification Kit (Thermo Fisher Scientific, Lithuania), according to the manufacturer’s instructions. DNA from fleas was extracted from each specimen individually using 2.5% ammonium hydroxide solution [61].

Nested PCR targeting the 16S–23S rRNA ITS region was performed using the external primers WITS-F (5-ACC TCC TTT CTA AGG ATG AT-3′) and WITS-R (5-CTC TTT CTT CAG ATG ATG ATCC-3′) and internal primers Bh311-332F (5′-CTC TTT CTT CAG ATG ATG ATCC-3′) and ITS-R (ITS)(5-GCG GTT AAG CTT CCA ATC ATA-3′), according to previously described protocols [62,63]. Negative controls consisting of sterile, double-distilled water added to the first PCR mix rather than DNA were included after every five experimental samples.

PCR products were visualized on a 1.5% agarose gel (Thermo Fisher Scientific, Vilnius, Lithuania). *Bartonella*-positive samples selected for DNA sequencing were purified using the GeneJET™ Gel Extraction Kit (Thermo Fisher Scientific, Vilnius, Lithuania) and sequenced (Macrogen Europe company, Amsterdam, The Netherlands). 

The obtained sequences were edited and then aligned with each other and with *Bartonella* spp. 16S–23S rRNA ITS region sequences were registered in the GenBank database by using BLAST and MUSCLE computer algorithms implemented in the Mega X software package [64]. A phylogenetic tree was constructed using the maximum-likelihood (ML) method with the Tamura 3-parameter model and bootstrap analysis of 1000 replicates.

Partial 16S–23S rRNA ITS region sequences for representative samples obtained in this study were submitted to the GenBank database under the accession numbers MZ061900–MZ061911 and MZ061922–MZ061927.

### 4.3. Statistical Analysis

A Chi-square test (Statistica for Windows, version 7.0, Statsoft, Tulsa, OK, USA) was used to compare the prevalence of *Bartonella* in cats and cat fleas. The observed differences were considered to be significant when *p* < 0.05.

## Figures and Tables

**Table 1:** *Bartonella* spp. in cats and cat fleas confirmed based on PCR and sequencing.

Cats/Fleas		n ^1^	*B. henselae*	*B. clarridgeiae*	*B. schoenbuchensis*-Like	Total
Stray cats	<1-year-old	9	1	0	1	2
	>1-year-old	9	2	1	0	3
Pet cats	<1-year-old	11	1	0	0	1
	>1-year-old	134	0	1	1	2
Total		163	4	2	2	8
*Ct. felis*	male	24	2	0	1	3
	female	68	15	3	4	22
*Ct. canis*	male	4	0	0	2	2
	female	6	2	0	1	3
Total		102	19	3	8	30

^1^ number of tested samples.

**Table 2:** Characteristics of *Bartonella* infection in cats.

	Age		Sex	Clinical Symptoms	*Bartonella* spp.
Stray cats	<1-year-old	5 months	♀	No symptoms	*B. schoenbuchensis*-like
		6 months	♀	Corneal ulcer	*B. henselae*
	>1-year-old	6 years	♀	No symptoms	*B. henselae*
		3 years	♂	No symptoms	*B. henselae*
		4 years	♂	Diarrhea	*B. clarridgeiae*
Pet cats	<1-year-old	8 months	♀	Lethargy	*B. henselae*
	>1-year-old	10 years	♀	RBC and haemoglobin levels low	*B. schoenbuchensis*-like
		8 years	♂	no symptoms	*B. clarridgeiae*

♀—female; ♂—male.

**Table 3:** Detection of *Bartonella* in cat fleas.

Cats	No of *Bartonella* Positive/No of Tested Fleas (%)	*B. henselae*	*B. clarridgeiae*	*B. schoenbuchensis*-Like
1	17/34 (50%)	11	2	4
2	1/16 (6.3%)	1	0	0
3	6/14 (42.9%)	3	0	3
4	3/12 (25%)	2	0	1
5	2/5 (40%)	1	1	0
6	1/3 (33.3%)	1	0	0

**Table 4:** Differences in the 16S–23S rRNA ITS nucleotide sequences among *Bartonella henselae* strains from Lithuania and other countries. Sequences detected in this study are in bold. The number of samples represented by the sequence is given in parentheses (n = x).

GenBank Accession Numbers	Nucleotide Positions										Strain	Geographic Origin
	66	276	343	429	496	628	652	707	744	776		
CP020742; BX897699; L35101	C	A	C	T	C	A	T	G	T	T	Houston	Germany, Brazil, France
AF312496; **MZ061902 (n = 5), MZ061903 (n = 5 ), MZ061904, MZ061906, MZ061907 (n = 5), MZ061908, MZ061909, MZ061910**	G	A	C	T	C	A	T	G	T	T	URBHLIE 9	France, Lithuania
**MZ061905 (n = 2)**	G	A	T	C	C	A	T	G	T	T		Lithuania
**MZ061911**	G	A	C	T	T	G	T	A	T	T		Lithuania
AF369527	G	A	C	T	T	G	A	A	T	T	CAL-1	France
JQ009430	G	A	C	T	C	G	T	G	T	T	Q5BJ-CW	China
AF369528	G	C	C	T	C	G	A	G	T	T	90-615	France
AF312495	G	A	C	T	C	A	T	G	C	C	URBHLLY 8	France

## Data Availability

Data are contained within the article.
